# Supplementary material for: Killing of a Multispecies Biofilm Using Gram-Negative and Gram-Positive Targeted Antibiotic Released from High Purity Calcium Sulfate Beads
Source: Microorganisms. 2023 Sep 12;11(9):2296. doi: 10.3390/microorganisms11092296 (PMC10538001; doi:10.3390/microorganisms11092296)
Supplement: Supplementary file 1 [file microorganisms-11-02296-s001.zip › microorganisms-2502747-SI.pdf]

SUPPLEMENTAL MATERIAL
Killing of a Multispecies Biofilm Using Gram-Negative and Gram-Positive Targeted Antibiotic Released from High Purity Calcium Sulfate Beads

Kelly Moore [1], Anthony Li [1], Niraj Gupta [1], Tripti Thapa Gupta [1], Craig Delury [2], Sean S. Aiken [2], Phillip A. Laycock [2] and Paul Stoodley [1,3,4,5,6,*]

[1] Department of Microbial Infection and Immunity, The Ohio State University, Columbus, OH 43210, USA; kelly.e.moore20@gmail.com (K.M.); li.5906@osu.edu (A.L.); gupta.1107@osu.edu (N.G.); thapatripti.bme@gmail.com (T.T.G.)

[2] Biocomposites Ltd., Keele Science Park, Keele, Staffordshire ST5 5NL, UK; craig_delury@hotmail.com (C.D.); sa@biocomposites.com (S.S.A.); pl@biocomposites.com (P.A.L.)

[3] Department of Orthopedics, The Ohio State University, Columbus, OH 43203, USA

[4] Department of Microbiology, The Ohio State University, Columbus, OH 43210, USA

[5] National Centre for Advanced Tribology at Southampton (nCATS), Department of Mechanical Engineering, University of Southampton, Southampton SO17 1BJ, UK

[6] National Biofilm Innovation Centre (NBIC), University of Southampton, Southampton SO17 1BJ, UK

* Correspondence: paul.stoodley@osumc.edu; Tel.: +1-614-292-7826

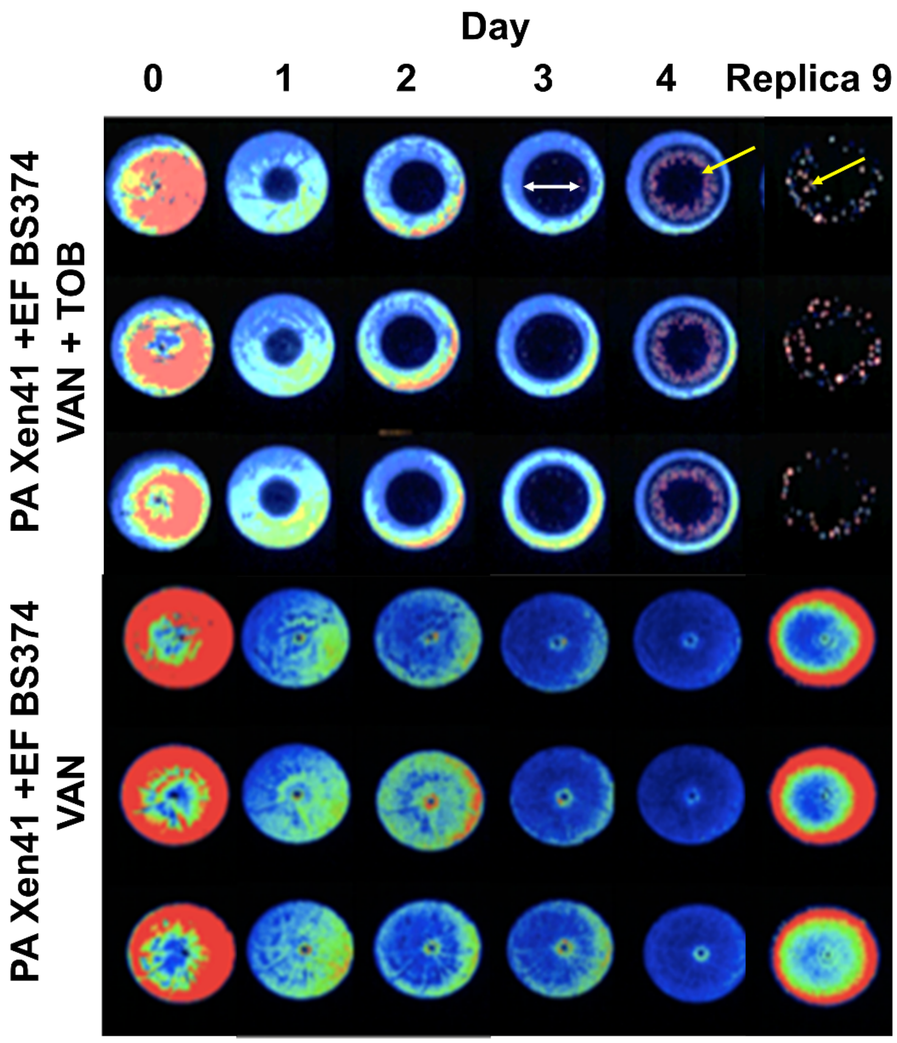

Figure S1. Bioluminescence image of PAXEN41 in a mixed PAXEN41 and EFBS374 agar plate biofilms treated with a bead loaded with either VAN and TOB together or VAN alone. Red = high signal, blue = low signal, black = no signal. Rows for each treatment are triplicate plates. For the VAN + TOB plate a zone of killing (ZOK) increased over time (white arrow) and at day 4 antibiotic tolerant variants grew within this zone (yellow arrow), allowing the PA ZOK and PA tolerant variants within the ZOK to be quantified. Replica plating of the day 9 plate shows a small popualtion of viable cells at the periphery while the area adjacent to the bead has been totally cleared of PA. For the VAN only plate there was some loss of lumiescence adjacent to the bead. The general dimming (red to black) over time is due to nutrient

depletion. The replica plate shows that PA in the biofilm was reduced by VAN in a zone adjacent to the bead.
